# Characterization of dysregulated lncRNA-mRNA network based on ceRNA hypothesis to reveal the occurrence and recurrence of myocardial infarction

**DOI:** 10.1038/s41420-018-0036-7

**Published:** 2018-02-21

**Authors:** Guangde Zhang, Haoran Sun, Yawei Zhang, Hengqiang Zhao, Wenjing Fan, Jianfei Li, Yingli Lv, Qiong Song, Jiayao Li, Mingyu Zhang, Hongbo Shi

**Affiliations:** 1 Department of Cardiology, The Fourth Affiliated Hospital of Harbin Medical University, 150001 Harbin, Heilongjiang China; 2 College of Bioinformatics Science and Technology, Harbin Medical University, 150081 Harbin, Heilongjiang China; 3 Emergency Cardiovascular Medicine, Inner Mongolia Autonomous Region People’s Hospital, Inner Mongolia Autonomous Region, 010017 Hohhot, China

## Abstract

Accumulating evidence has demonstrated that long non-coding RNAs (lncRNAs) acting as competing endogenous RNAs (ceRNAs) play important roles in initiation and development of human diseases. However, the mechanism of ceRNA regulated by lncRNA in myocardial infarction (MI) remained unclear. In this study, we performed a multi-step computational method to construct dysregulated lncRNA-mRNA networks for MI occurrence (DLMN_MI_OC) and recurrence (DLMN_MI_Re) based on “ceRNA hypothesis”. We systematically integrated lncRNA and mRNA expression profiles and miRNA-target regulatory interactions. The constructed DLMN_MI_OC and DLMN_MI_Re both exhibited biological network characteristics, and functional analysis demonstrated that the networks were specific for MI. Additionally, we identified some lncRNA-mRNA ceRNA modules involved in MI occurrence and recurrence. Finally, two new panel biomarkers defined by four lncRNAs (*RP1-239B22.5*, *AC135048.13*, *RP11-4O1.2*, *RP11-285F7.2*) from DLMN_MI_OC and three lncRNAs (*RP11-363E7.4*, *CTA-29F11.1*, *RP5-894A10.6*) from DLMN_MI_Re with high classification performance were, respectively, identified in distinguishing controls from patients, and patients with recurrent events from those without recurrent events. This study will provide us new insight into ceRNA-mediated regulatory mechanisms involved in MI occurrence and recurrence, and facilitate the discovery of candidate diagnostic and prognosis biomarkers for MI.

## Introduction

Myocardial infarction (MI) is one of the most serious types of coronary artery disease, which often lead to myocardial cell death due to prolonged ischemia^1^. It is a leading cause of morbidity, mortality, and cost to society^2^. The recurrence of MI following first-time occurrence will make coronary artery conditions more severely and greatly increase the risk of death in patients^3^. Thus strategies for prediction of recurrent events will prolong survival in post-MI patients^4^. Non-high-density lipoprotein cholesterol value was recently found to be a strong predictor of recurrent MI^5^, and phospholipase A2 expression in coronary thrombus has been reported to be related with recurrence of cardiac events after MI^6^. Although some biochemical markers such as cardiac troponins T and I, creatine kinase-MB are clinically used for diagnosis of MI, they only indicate myocardial damage, and the molecular mechanisms underlying MI and recurrence of MI are not reflected.

There have been numerous researches documented that less than 2% of the human genome encodes protein-coding genes, and non-coding RNAs (ncRNAs) constitute most of the human transcriptome^7^. ncRNAs include short ncRNAs and long ncRNAs. microRNAs (miRNAs) are a class of important short ncRNAs with approximately 22 nucleotides in length, which have been extensively studied. It mainly inhibit gene expression by binding to the 3′ untranslated regions of target mRNAs^8^. miRNAs have been reported to be implicated in numerous diseases^9, 10^, including MI^11^. While long non-coding RNAs (lncRNAs) represent a major class of ncRNAs, with greater than 200 nucleotides in length, which could regulate genes at transcriptional, post-transcriptional, and epigenetic levels^12^. The dysregulation of lncRNA expression is therefore associated with various diseases^13^, including cancers^14, 15^, neurodegeneration diseases^16^, and cardiovascular diseases^17, 18^. For example, knockdown of a novel lncRNA, Mirt1, was recently reported to improved cardiac functions, decreased cardiomyocytes apoptosis, and attenuated inflammatory cell infiltration in cardiac fibroblasts in acute MI mice^19^. In rat cardiac muscle H9c2 cells, downregulation of lncRNA KCNQ1OT1 has been found to prevent myocardial ischemia/reperfusion injury following acute MI^20^. However, little is known about lncRNAs in MI.

Theoretical and experimental studies have demonstrated that a large number of miRNA-binding sites exist on different types of RNA transcripts, indicating that diverse RNA transcripts containing the miRNA-binding sites can regulate each other through competing for shared miRNAs, thus acting as competing endogenous RNAs (ceRNAs)^21–23^. Importantly, lncRNAs could compete with miRNA target mRNAs for miRNA molecules, and thus regulate miRNA-mediated target repression^21, 22^. This type of ceRNA crosstalk has been widely observed in different biological processes and diseases. For example, a lncRNA (lnc-mg) that is specifically enriched in skeletal muscle was recently identified, and it was found to promote myogenesis by acting as a ceRNA for miR-125b to affect protein abundance of insulin-like growth factor 2^24^. Additionally, lncRNA MIAT was demonstrated to function as a ceRNA to upregulate DAPK2 by regulating miR-22-3p in diabetic cardiomyopathy^25^. However, ceRNA mechanisms associated with MI have not been investigated.

**Fig. 1:**
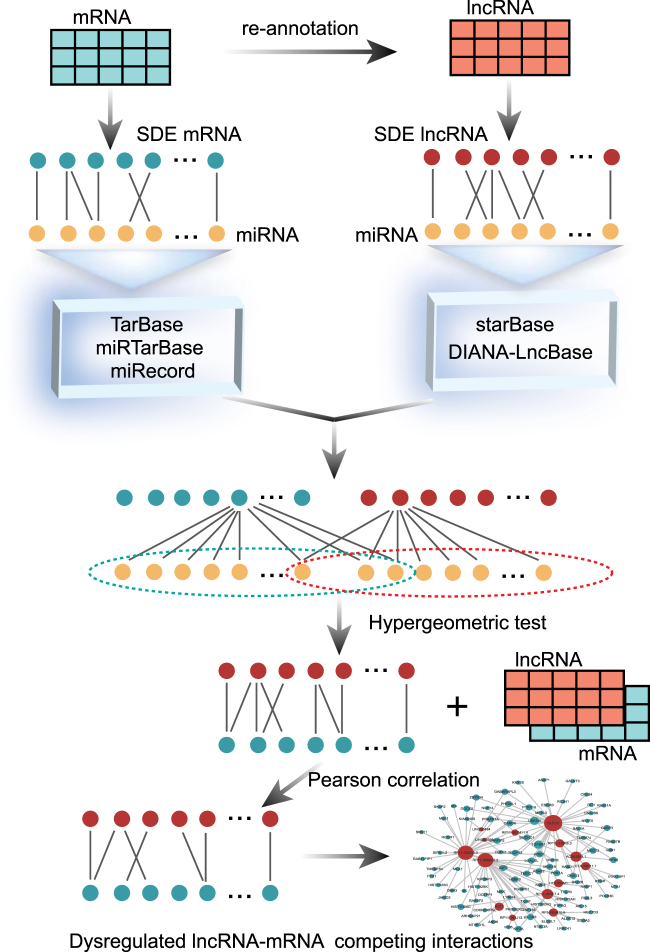
An integrative pipeline to construct the dysregulated lncRNA-mRNA ceRNA network for MI occurrence and recurrence. First, starting from significantly differentially expressed (SDE) mRNAs and lncRNAs, experimentally verified miRNA–mRNA and miRNA–lncRNA interaction relationships were integrated, and hypergeometric test (*p*-value < 0.05) was performed to identify candidate lncRNA–mRNA competing interactions. Second, the Pearson correlation coefficient (PCC) was further utilized to screen mRNA–lncRNA competing interactions by using expression profiles. Finally, dysregulated lncRNA-mRNA ceRNA network for MI occurrence and recurrence were constructed by merging the dysregulated lncRNA–mRNA ceRNA interactions, respectively

In this study, we systematically integrated regulatory interactions among lncRNAs, miRNAs and mRNAs and expression profile data, and identified lncRNA–mRNA competing interactions in MI based on “ceRNA hypothesis”. We then constructed dysregulated lncRNA-mRNA networks for MI occurrence (DLMN_MI_OC) and recurrence (DLMN_MI_Re). The pipeline of construction the network was shown in Fig. 1. Based on the two constructed networks, we intended to investigate: (1) topological properties and biological functions of DLMN_MI_OC and DLMN_MI_Re, (2) lncRNA-mRNA ceRNA modules in DLMN_MI_OC and DLMN_MI_Re, (3) potential lncRNA biomarkers for MI occurrence and recurrence.

## Results

### Differentially expressed mRNAs and lncRNAs

After pre-processing of the gene expression profiles, expression profile data of 21,695 mRNAs and 1542 lncRNAs were retained for further studied. We then compared the expression profiles of mRNAs and lncRNAs between MI patients and controls using the R “limma” package, and 1001 significantly differentially expressed (SDE) mRNAs and 55 SDE lncRNAs were identified (*p* < 0.01). Among these, 437 mRNAs and 34 lncRNAs were upregulated and 564 mRNAs and 21 lncRNAs were downregulated. The expression profiles of mRNAs and lncRNAs between MI patients with recurrent events and those without recurrent events were also analyzed using the R “limma” package. Considering the limited number of MI patients with recurrent events, the mRNAs and lncRNAs with *p* < 0.05 were selected as SDE mRNAs and lncRNAs. Thus, 859 SDE mRNAs and 60 SDE lncRNAs were obtained. Of these, 538 mRNAs and 39 lncRNAs were upregulated and 321 mRNAs and 21 lncRNAs were downregulated.

### Construction of the DLMN_MI_OC and DLMN_MI_Re

**Fig. 2:**
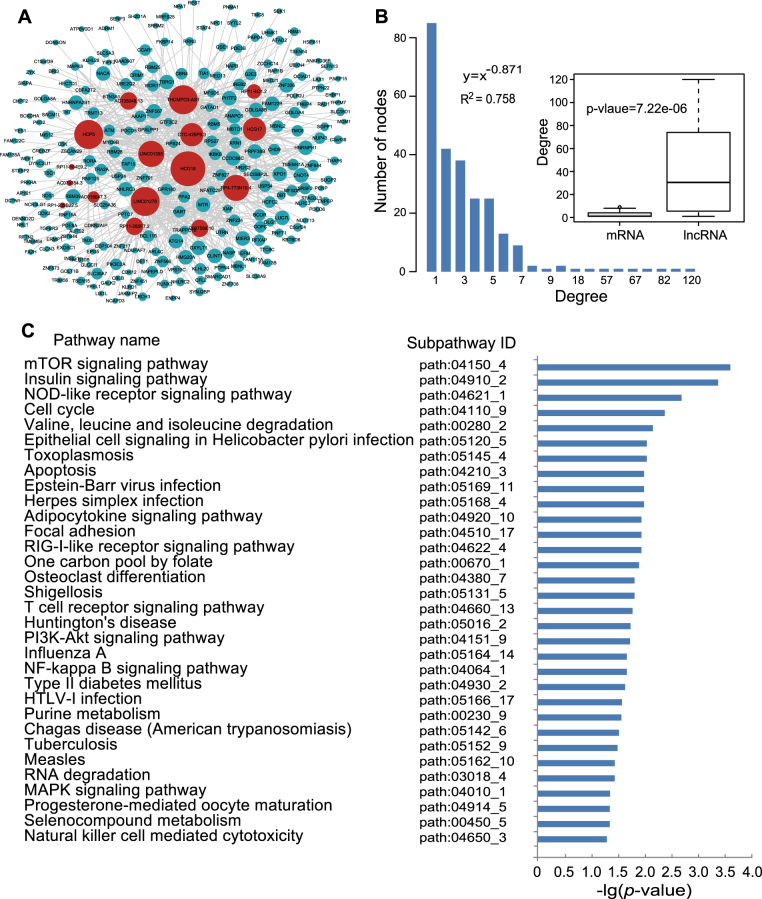
DLMN_MI_OC and its structural and functional features. **a** Global view of the DLMN_MI_OC for MI occurrence. The DLMN_MI_OC consists of 660 edges between 235 mRNAs (green circles) and 16 lncRNAs (red circles). The size of nodes is proportional to the degrees of the nodes in the network. **b** Degree distribution of nodes in the DLMN_MI_OC. Degree of lncRNAs is signifcantly higher than that of mRNAs. **c** Significantly enriched KEGG subpathways (cancer subpathways removed) of mRNAs in the network

**Fig. 3:**
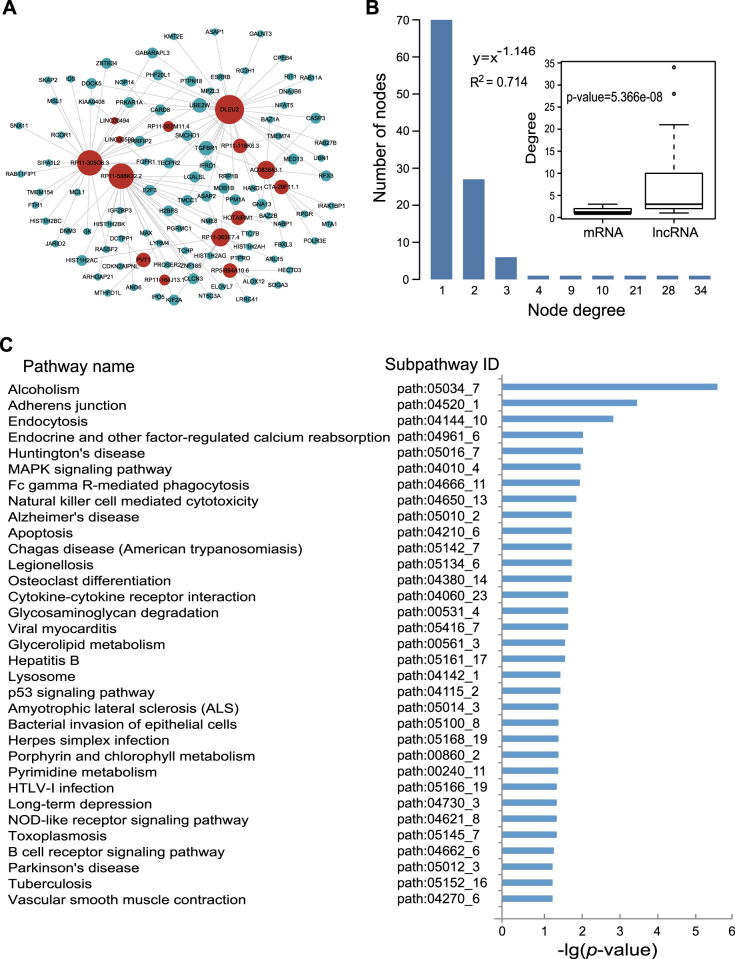
DLMN_MI_Re and its structural and functional features. **a** Global view of the DLMN_MI_Re for MI recurrence. The DLMN_MI_Re consists of 124 edges between 95 mRNAs (green circles) and 14 lncRNAs (red circles). The size of nodes is proportional to the degrees of the nodes in the network. **b** Degree distribution of nodes in the DLMN_MI_Re. Degree of lncRNAs is significantly higher than that of mRNAs. **c** Significantly enriched KEGG subpathways (cancer subpathways removed) of mRNAs in the network

The DLMN_MI_OC and DLMN_MI_Re were both constructed based on ceRNA theory by integrating expression profile data and regulatory relationships of mRNAs, miRNAs, and lncRNAs. As described in “Materials and methods”, an lncRNA–mRNA competing interaction pair was selected if the lncRNA and the mRNA significantly shared common miRNAs, and the expression of the lncRNA and the mRNA is positively correlated. As a result, we identified 660 and 124 dysregulated lncRNA-mRNA pairs for MI occurrence and recurrence, respectively (Supplementary Table 1). By merging these dysregulated interactions, the DLMN_MI_OC and DLMN_MI_Re were constructed. As demonstrated in Figs. 2a and 3a, the DLMN_MI_OC included 251 nodes (235 mRNAs and 16 lncRNAs) and 660 ceRNA interactions, and the DLMN_MI_Re contained 109 nodes (95 mRNAs and 14 lncRNAs) and 124 ceRNA interactions.

### Topological and biological functional analysis of the DLMN_MI_OC and DLMN_MI_Re

To investigate the global view of the DLMN_MI_OC and DLMN_MI_Re, we computed degree and degree distribution, which are basic topological features of biological networks, as shown in Figs. 2b, 3b, and Supplementary Table 2. For the DLMN_MI_OC, the average node degree of mRNAs and lncRNAs was 2.81 (range from 1–9) and 41.25 (range from 1–120), respectively. While in the DLMN_MI_Re, the average node degree of mRNAs and lncRNAs was 1.31 (range from 1–3) and 8.86 (range from 1–34), respectively. Moreover, degree of lncRNAs is signifcantly higher than that of mRNAs (Wilcoxon rank-sum test) both in the DLMN_MI_OC and DLMN_MI_Re. The degree distribution of the DLMN_MI_OC and DLMN_MI_Re were both significantly right-skewed, demonstrating that only a small portion of nodes highly connected with other nodes, and had a significantly higher degree. These nodes were usually considered as hub nodes.

We paid close attention to the hub nodes, which have been demonstrated to play critical roles in maintaining the overall connectivity of the network. According to recent studies, the nodes with the highest (top 5%) degree were selected as hubs^26–28^. As a result, 14 hub nodes in the DLMN_MI_OC were obtained, including 11 lncRNAs (HCG18, THUMPD3-AS1, LINC01278, HCP5, RP4-773N10.4, LINC01355, HCG17, CTC-429p9.3, RP11-285F7.2, AC007566.10, and AC135048.13) and 3 mRNAs (XPO1, TAF15, and GXYLT1). Among these 11 lncRNAs, RP11-285F7.2 has recently been reported to be differentially expressed in induced pluripotent stem cell cardiomyocytes following treatment with trastuzumab^29^, and much of these lncRNAs have been found to be associated with cancers. Similarly, six hub nodes in the DLMN_MI_Re were identified, and they were all lncRNAs (DLEU2, RP11-30506.3, RP11-588K22.2, RP11-363E7.4, AC083843.1, and PVT1). Among them, DLEU2 was found to be contained in a deletion at chr13q14.3 in an earthquake-associated stress cardiomyopathy case, and this region including a gene play important roles in regulating voltage-gated potassium channel activity^30^. PVT1 was shown to be differentially expressed between MI and sham-operated mice in a recently published study^31^.

We further examined the biological function of the DLMN_MI_OC and DLMN_MI_Re. Significantly enriched KEGG biological subpathways were identified by applying SubpathwayMiner^32^ using 235 mRNAs in the DLMN_MI_OC and 95 mRNAs in the DLMN_MI_Re. Consequently, 36 and 43 significant subpathways were obtained with a *p*-value of <0.05 (Supplementary Table 3). To more clearly demonstrate the results, cancer pathways were removed, as demonstrated in Figs. 2c and 3c. For the DLMN_MI_OC, several pathways well known in MI were significantly enriched, such as NOD-like receptor signaling pathway, T cell receptor signaling pathway, PI3K-Akt signaling pathway, NFκB signaling pathway, MAPK signaling pathway, and apoptosis, indicating inflammation, immune response, and cell apoptosis. Additionally, some other pathways were also closely related with MI, including mTOR signaling pathway and focal adhesion. For the DLMN_MI_Re, we found that several pathways were cardiovascular-related pathways, such as viral myocarditis and vascular smooth muscle contraction, and some pathways played important roles in MI, including MAPK signaling pathway, NOD-like receptor signaling pathway, B cell receptor signaling pathway, and apoptosis. Meanwhile, pathways associated with emotion and diet were also significantly enriched, such as alcoholism pathway and long-term depression pathway. It is noteworthy that, among all the pathways, the *p*-value of alcoholism pathway was the most significant. All these enriched pathways will provide us important cellular process information for understanding molecular pathology and recurrence of MI.

### lncRNA-mRNA ceRNA modules in MI occurrence and recurrence

To further study ceRNA crosstalks between mRNAs and lncRNAs in MI occurrence and recurrence, lncRNA-mRNA ceRNA modules were identified. We performed hierarchical clustering on DLMN_MI_OC and DLMN_MI_Re using Cluster3 software by the city-block distance and complete linkage method (shown by JavaTreeView imaging software). As a result, three ceRNA network modules were identified in DLMN_MI_OC and DLMN_MI_Re, respectively.

**Fig. 4:**
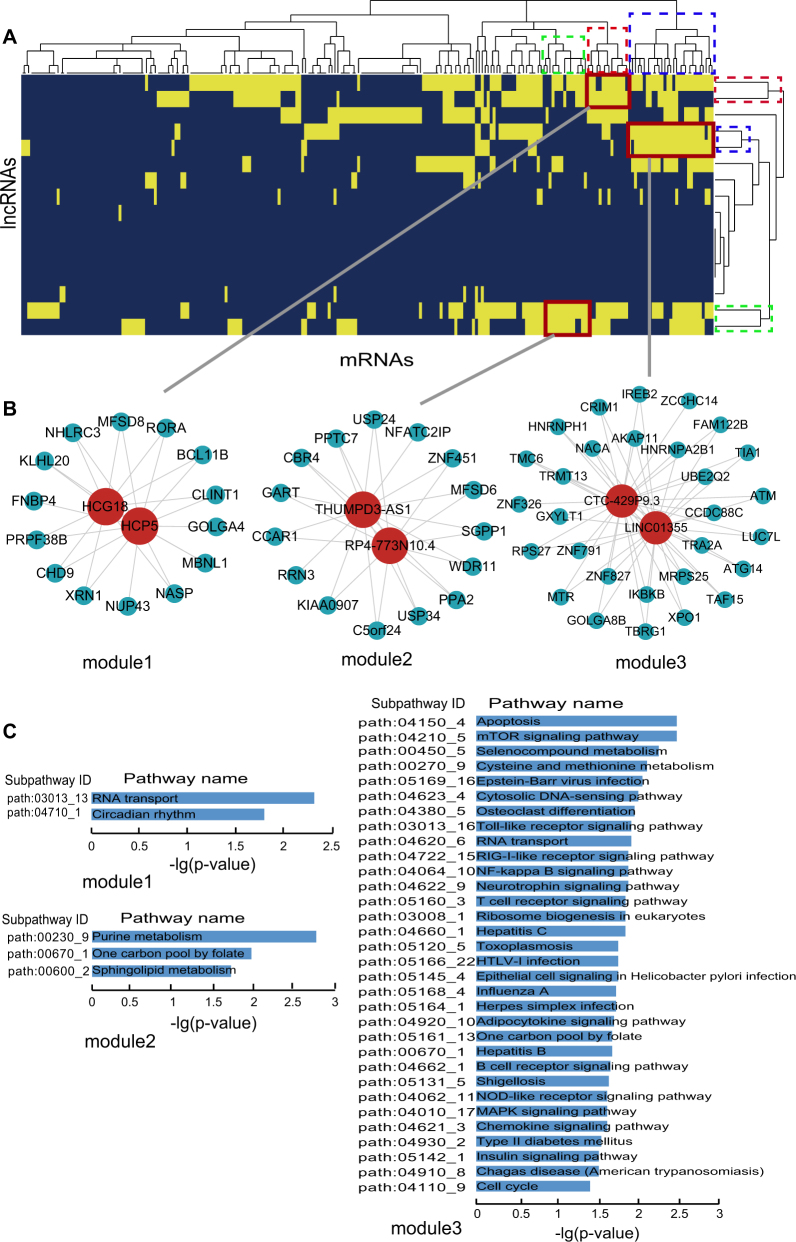
lncRNA-mRNA ceRNA modules in DLMN_MI_OC. **a** Hierarchical clustering of the DLMN_MI_OC for MI occurrence. **b** Three identified lncRNA-mRNA ceRNA modules in the DLMN_MI_OC. **c** Subpathway enrichment analysis of the three ceRNA modules

lncRNA-mRNA ceRNA modules in DLMN_MI_OC were shown in Fig. 4 and Supplementary Table 4. In the first module, lncRNA HCG18 competed with 14 mRNAs and another lncRNA HCP5. In the second module, 2 lncRNAs (THUMPD3-AS1 and RP4-773N10.4) and 15 mRNAs competed with each other. While the third module contained 31 ceRNAs including 2 lncRNAs (CTC-429p9.3 and LINC01355) and 29 mRNAs. We further investigated the biological function of each module, and significant enriched KEGG subpathways were identified using mRNAs in each module (Supplementary Table 4). In module 1, RNA transport and circadian rhythm were significantly enriched. Experimental and clinical evidences have suggested that the onset of MI, infarct size, healing, and cardiac function after MI all exhibited a similar time-of-day dependency^33–35^. In module 2, purine metabolism, one-carbon pool by folate and sphingokupid metabolism were statistically enriched. Simultaneously, metabolites involved in purine metabolism has been reported to be potential pathological biomarkers related to isoproterenol-induced MI^36^. DNA-methylation patterns in specific regions of the one-carbon metabolism and the homocysteine pathway genes regulated MI risk conferred by folate and B-vitamins low intake^37^. Additionally, sphingolipid-mediated cell signaling played important roles in acute MI and heart failure^38^. Module 3 is the largest one, and the enrichment results were highly related to MI.

**Fig. 5:**
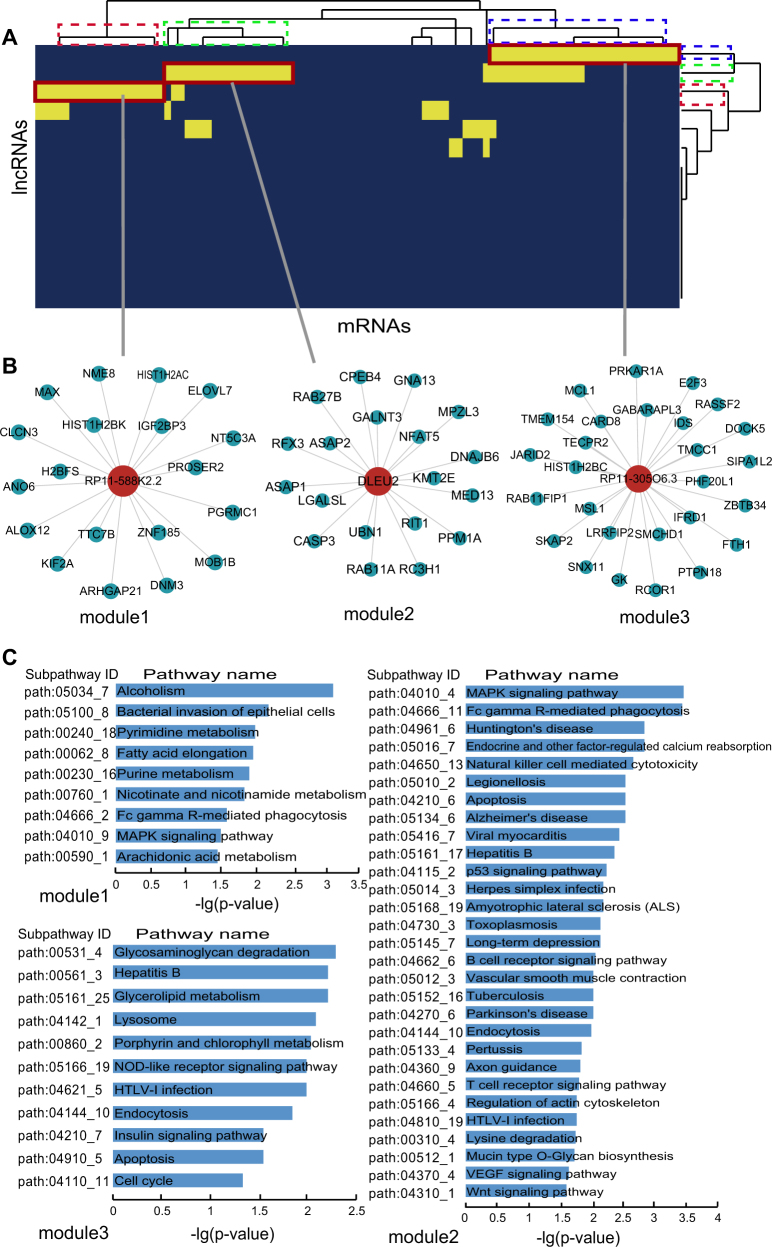
lncRNA-mRNA ceRNA modules in DLMN_MI_Re. **a** Hierarchical clustering of the DLMN_MI_Re for MI recurrence. **b** Three identified lncRNA-mRNA ceRNA modules in the DLMN_MI_Re. **c** Subpathway enrichment analysis of the three ceRNA modules

lncRNA-mRNA ceRNA modules in DLMN_MI_Re were shown in Fig. 5 and Supplementary Table 4. The first module and the second module both included 20 ceRNAs (19 mRNAs and 1 lncRNA), and the third module contained 28 ceRNAs (27 mRNAs and 1 lncRNA). As can be seen from the enrichment results, the pathways were closely related to MI or MI recurrence. Alcoholism was the most significantly enriched pathway in module 1, and most pathways in this module were metabolism pathways, such as lipid metabolism (arachidonic acid metabolism and fatty acid elongation) and nucleotide metabolism (purine metabolism and pyrimidine metabolism). MRNAs in module 2 enriched the most pathways including viral myocarditis, long-term depression, and vascular smooth muscle contraction. The enrichment results of module 3 were also related to MI.

### Identification of candidate lncRNA biomarkers for MI occurrence and recurrence

**Fig. 6:**
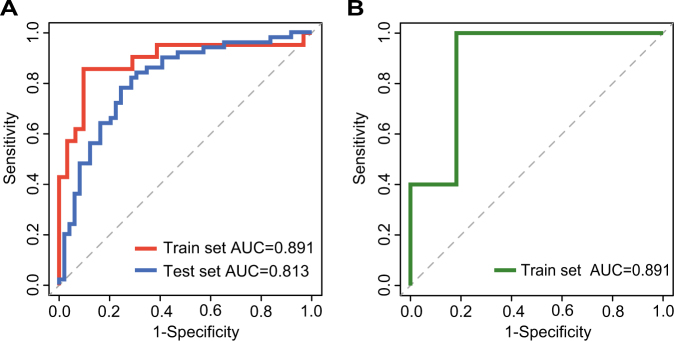
Classification performance of the identified lncRNA biomarkers for MI occurrence and recurrence based on LOOCV analysis. **a** ROC curves and AUC values of lncRNA biomarkers for MI occurrence. **b** ROC curves and AUC values of lncRNA biomarkers for MI recurrence

To identify candidate lncRNA biomarkers for MI occurrence and recurrence based on ceRNA mechanisms, we examined 16 lncRNAs in the DLMN_MI_OC and 14 lncRNAs in the DLMN_MI_Re. As described in the “Materials and methods”, 7 and 6 lncRNAs mostly related to MI occurrence and recurrence were, respectively, selected using random forest supervised classification algorithm in the training set. There were 2^7^−1 = 127 and 2^6^−1 = 63 combinations of these remaining lncRNAs. Finally, we computed classification accuracies for all these combinations using support vector machine (SVM) classification model, and the optimal lncRNA biomarkers were obtained. As a result, two panel biomarkers defined by four lncRNAs (*RP1-239B22.5*, *AC135048.13*, *RP11-4O1.2*, *RP11-285F7.2*) and three lncRNAs (*RP11-363E7.4*, *CTA-29F11.1*, *RP5-894A10.6*), with the highest classification accuracy, were identified for MI occurrence and recurrence, respectively. Detailed information was shown in Table 1. For the signature of four lncRNAs for MI occurrence, an accuracy of 0.885 and an AUC value of 0.891 were obtained in the training set (Fig. 6a) by applying leave-one-out cross-validation (LOOCV). The signature was further examined in an independent test set including 50 healthy controls and 49 acute MI patients, and an accuracy of 0.813 and an AUC value of 0.768 were achieved (Fig. 6a). In the same way, for the signature of three lncRNAs for MI recurrence, we obtained an accuracy of 0.889 and an AUC value of 0.891 in the training set by performing LOOCV (Fig. 6b). All the results demonstrated that the signatures we identified were accurate and reliable in distinguishing controls from patients, and patients with from those without recurrent events.

**Table 1:** The detailed information of the identified lncRNA biomarkers for MI occurrence and recurrence

Ensembl ID	Gene name	Chromosomal location	*p*-value	Known rearch
ENSG00000260196.1	RP1-239B22.5^a^	chr11:17,380,649-17,383,531(+)	0.0002	Severe pre-eclampsia^56^
ENSG00000261487.1	AC135048.13^a^	chr16:30,948,386-30,956,511(+)	0.0042	No
ENSG00000259953.1	RP11-4O1.2^a^	chr9:112,032,555-112,037,730(−)	0.0046	(1) Papillary thyroid carcinoma^57^ (2) Associated with height^58^
ENSG00000242861.1	RP11-285F7.2^a^	chr1:225,840,883-225,846,522(−)	0.0099	(1) Cardiomyocytes^29^ (2) Non-small cell lung cancer^59^
ENSG00000260912.1	RP11-363E7.4^b^	chr9:19,453,209-19,455,173(+)	0.0169	Gastric cancer^60^
ENSG00000260708.1	CTA-29F11.1^b^	chr22:46,761,894-46,762,563(−)	0.0231	Human adenovirus infected cells^61^
ENSG00000270157.1	RP5-894A10.6^b^	chr7:141,662,922-141,663,846(−)	0.0298	Head and neck squamous cell carcinoma^62^

^a^ Denotes the identified lncRNA biomarkers for MI occurrence

^b^ Indicates the identified lncRNA biomarkers for MI recurrence

We further examined the significantly enriched subpathways using mRNAs co-expressed with the lncRNA biomarkers for MI occurrence and recurrence (Supplementary Table 5). The results showed that several pathways responsing to wounding and inflammatory response, such as mTOR signaling pathway, PI3K-Akt signaling pathway, leukocyte transendothelial migration, were all associated with MI occurrence. For MI recurrence, only three subpathways were enriched. Among these, alcoholism pathway was the most significant one, and its *p*-value was much more smaller than the other pathways. These results suggested that the lncRNA biomarkers we identified played important roles in the process of occurrence and recurrence of MI.

## Discussion

Recent studies have revealed that ceRNAs including lncRNAs and mRNAs could mutually regulate each other via competing for their shared miRNAs, which are important for physiological and pathological processes of diseases. In the present study, based on ceRNA mechanisms, we systematically constructed two networks of DLMN_MI_OC and DLMN_MI_Re for MI ocurrence and recurrence by integrating genome-wide lncRNA and mRNA expression profile data and experimentally verified miRNA–target interactions. The two networks were both presented modular features and high functional specificity for MI. From the networks, two candidate panel biomarkers defined by four lncRNAs and three lncRNAs were identified for MI occurrence and recurrence, respectively.

Functional enrichment analysis using mRNAs co-expressed with lncRNAs revealed biological pathways associated with MI occurrence and recurrence. Interestingly, for MI recurrence, alcoholism pathway was the most significantly enriched one, and the *p*-value of this pathway was much more smaller than the other ones. This phenomenon was also observed when using three lncRNAs biomarker for MI recurrence. Heavy consumption of alcohol increased the risk of acute MI in the subsequent 24 h, particularly in older persons it has been reported^39^. Cardioembolic stroke patients with alcohol abuse increased the risk of early recurrent systemic embolization^40^, and alcoholism pathway significantly enriched in chronic phase of MI was found in our previous studies^41^. These results suggested that alcoholism might have relationships with MI recurrence, and MI patients may be likely to make dietary changes to prevent future infarcts. Simultaneously, long-term depression pathway was significantly enriched, and depression after MI increased the risk of mortality and cardiovascular events^42^. In addition, we found that cancer pathways were also significantly enriched, suggesting relationships between MI and the cancers. As is already reported, tumor invasion to the heart with tumor compression on the coronary arteries may cause MI^43^.

The DLMN_MI_OC and DLMN_MI_Re were constructed by using experimentally verified miRNA-mRNA and miRNA-lncRNA regulations. Here, we emphasized the credibility rather than the coverage. Thus, we did not employ predicted data. However, experimentally supported interactions were neither complete nor unbiased, and further experimental confirmation was needed. With an improvement of the quantity and quality of the miRNA–mRNA and miRNA–lncRNA interactions and sample matched expression profile data of mRNA, miRNA, and lncRNA, the dysregulated lncRNA-mRNA ceRNA pairs we identified for MI occurrence and recurrence will be more accurate.

In summary, our study provided a global view for ceRNA crosstalks between mRNAs and lncRNAs in MI occurrence and recurrence by constructing ceRNA networks, and we identified two candidate panel biomarkers defined by four lncRNAs and three lncRNAs for MI occurrence and recurrence. All the results will improve our understanding of molecular mechanisms underlying MI pathology and recurrence from ceRNA perspective, and help us to discover true biomarkers for MI occurrence and recurrence.

## Materials and methods

### Gene expression profiles

The MI-related gene expression profiles of GSE48060 based on Affymetrix Human Genome U133 Plus 2.0 Array were downloaded from the publicly available Gene Expression Omnibus database (https://www.ncbi.nlm.nih.gov/geo/query/acc.cgi?acc=GSE48060)^44^, which included 21 normal controls and 31 acute MI patients. All these patients conducted a 18-month follow-up. As a result, five patients with recurrent events and 22 without any recurrent events.

### Acquisition of lncRNA expression profiles

The microarray gene expression data was normalized using the RMA algorithm and log2 transformed. To obtain the corresponding lncRNA expression profile data, we re-annotated the probes in the HG-U133_Plus_2.0 array to lncRNAs, according to previous studies^14, 45, 46^. First, we re-mapped the probes (probe sets) of Affymetrix HG-U133_Plus_2.0 array to the human genome (GRCh38) using SeqMap^47^. The probes (probe sets) were retained when they were uniquely mapped to the human genome with no mismatch. Second, we matched the chromosomal position of the above probes (probe sets) to the chromosomal position of lncRNAs from the GENCODE project (http://www.gencodegenes.org, release 25)^7^. Finally, we obtained lncRNA expression profiles including 1542 lncRNAs.

### Expression profiles analysis

For mRNA expression profiles and lncRNA expression profiles, if multiple probes mapping to the same gene, the expression values were averaged. We then retained protein-coding genes in mRNA expression profiles. SDE mRNAs and lncRNAs between MI patients and control subjects were identified using empirical Bayesian method implemented in R “limma” package^48^. The genes with *p* < 0.01 were considered as SDE genes. Considering the limited number of MI patients with recurrent events, the SDE mRNAs and lncRNAs between MI patients with recurrent events and those without recurrent events were selected with *p* < 0.05.

### miRNA–mRNA and miRNA–lncRNA interaction data

The experimentally verified miRNA–mRNA interaction relationships were downloaded from TarBase (version 6.0)^49^, miRTarBase (version 6.1)^50^, and miRecords (version 4)^51^ databases. By integrating the above three databases, we obtained 359,591 non-redundant miRNA–mRNA interactions. The experimentally validated miRNA–lncRNA interactions were extracted from starBase v2.0^52^ and DIANA-LncBase v2.0^53^, after removing repeating miRNA-lncRNA entries, 64,716 miRNA–lncRNA relationships were retained.

### Hypergeometric test

To identify candidate mRNA–lncRNA competing interaction pairs that shared the same miRNAs, a *p*-value was calculated using cumulative hypergeometric test based on the common miRNAs of any pair of mRNAs and lncRNAs. The formula was as follows:

$$p = \mathop {\sum}\nolimits_{i = |N_{\mathrm{mRNA}}| \cap |N_{\ln c}|}^{\min (|N_{\mathrm{mRNA}}|,|N_{\ln c}|)} {\frac{{\left( {\begin{array}{*{20}{c}} {|N_{\mathrm{mRNA}}|} \\ i \end{array}} \right)\left( {\begin{array}{*{20}{c}} {\mathrm{Total} - |N_{\mathrm{mRNA}}|} \\ {|N_{\ln c}| - i} \end{array}} \right)}}{{\left( {\begin{array}{*{20}{c}} {\mathrm{Total}} \\ {|N_{\ln c}|} \end{array}} \right)}}},$$

where *N*_mRNA_ is the number of miRNAs targeted a given mRNA, while *N*_In *c*_ is the number of miRNAs regulated a given lncRNA and Total is the number of common miRNAs between all human miRNAs targeted human mRNAs and all human miRNAs regulated all human lncRNAs. The mRNA-lncRNA competing pairs with a *p*-value less than 0.05 were selected as significant pairs.

### Dysregulated lncRNA–mRNA competing interactions

The dysregulated mRNA–lncRNA competing interactions for MI occurrence and recurrence were identified based on “ceRNA hypothesis”. This process comprised two steps as follows (Fig. 1). First, starting from SDE mRNAs and lncRNAs, experimentally verified regulatory relationships of miRNA-mRNA and miRNA-lncRNA were identified as described above. We then performed hypergeometric test to test the significance of shared miRNAs between lncRNA and mRNA pairs. A miRNA and an lncRNA pair was considered as a candidate lncRNA–mRNA competing interaction if the *p*-value of hypergeometric test was less than 0.05. Second, we further screened mRNA–lncRNA competing interactions using expression profiles. The Pearson correlation coefficient (PCC) was utilized to evaluate expression correlation between mRNAs and lncRNAs. An mRNA–lncRNA competing interaction was defined if the PCC of the mRNA and the lncRNA were positively correlated. In order to increase the reliability of the results, we retained the top correlated lncRNA-mRNA pairs for further analysis according to previous studies^28, 54^. The PCC of these mRNA-lncRNA pairs are higher than the threshold of the 95th percentile of the corresponding overall correlation distribution (PCC > 0.570 for MI occurrence, and PCC > 0.564 for MI recurrence, Supplementary Table 1).

### Network generation, analysis, and functional evaluation

The dysregulated lncRNA-mRNA network based on “ceRNA hypothesis” for MI occurrence (DLMN_MI_OC) and recurrence (DLMN_MI_Re) was constructed by merging the dysregulated lncRNA–mRNA interactions identified above.

To assess network characteristics, we computed degree of each node in the network, and analyzed their degree distribution. Degree of a node is the most elementary feature of network, and it is defined the number of edges linked to it. If degree distribution of a given network follows a power law, the network would have only a few nodes with a large number of edges (i.e., hubs). Hub nodes in a network were selected as the highest (top 5%) degree according to previous studies^26–28^.

We implemented enrichment analysis of mRNAs in the network to assess the biological function of a network. Significantly enriched KEGG subpathways were identified using the R “SubpathwayMiner” package^32^. A KEGG subpathway with *p* < 0.05 was considered as significantly enriched.

### Classification of lncRNA biomarkers for MI occurrence and recurrence

To evaluate the classification efficiency of lncRNA biomarkers in distinguishing controls from patients and patients with recurrent events from those without recurrent events, a classification model based on SVM was implemented using the R “e1071” package, and the performance was estimated by classification accuracy and the area under the receiving operating curve (AUC). An AUC value ranges from 0 to 1, with 0.5 implying randomly obtained performance and 1.0 indicating perfect predictive performance.

First, lncRNAs mostly related to MI occurrence and recurrence were selected using random forest supervised classification algorithm, according to a recently published study^55^. At each step, each lncRNA was estimated an important score using the out-of-bag samples through permutation test, and 1/3 less important lncRNAs were discarded. Second, we reserved certain lncRNAs considering a balance between classification accuracy and the number of lncRNAs. Finally, classification accuracy for all combinations of the remaining lncRNAs was evaluated by applying SVM, and the optimal lncRNA biomarkers were selected.

The lncRNA profiles obtained by probe re-annotation of GSE48060 were used as training set. On the same microarray platform, we found another MI-related gene expression profile data of GSE66360, and the corresponding lncRNA profiles were obtained by implementing the same method. The dataset of GSE66360 included 50 healthy samples and 49 acute MI patients, but it did not contain the information about recurrent event. Therefore, the dataset was used as an independent test set for MI occurrence.

## Electronic supplementary material

Supplementary Table 1

Supplementary Table 2

Supplementary Table 3

Supplementary Table 4

Supplementary Table 5

Supplementary Information
