# Supplementary material for: Characterization of dysregulated lncRNA-mRNA network based on ceRNA hypothesis to reveal the occurrence and recurrence of myocardial infarction
Source: Cell Death Discov. 2018 Feb 21;4:35. doi: 10.1038/s41420-018-0036-7 (PMC5841419; doi:10.1038/s41420-018-0036-7)
Supplement: Supplementary file 6 — Supplementary Information [file 41420_2018_36_MOESM6_ESM.docx]

# Supplementary Information

## Supplementary Table 1. Dysregulated lncRNA-mRNA competing interactions for MI occurrence and recurrence.

## Supplementary Table 2. Degree of each node in DLMN_MI_OC and DLMN_MI_Re.

## Supplementary Table 3. Significantly enriched subpathways of DLMN_MI_OC and DLMN_MI_Re.

## Supplementary Table 4. LncRNA-mRNA ceRNA modules in DLMN_MI_OC and DLMN_MI_Re and significantly enriched subpathways of each module.

## Supplementary Table 5. Significantly enriched subpathways of the identified lncRNA biomarkers for MI occurrence and recurrence.
